# Molecular Mechanisms and Antioxidant Effects of *Latilactobacillus sakei* F1, *Lacticaseibacillus paracasei* D2, *Lacticaseibacillus rhamnosus* JL, and *Weissella cibaria* JLK Isolated from Spontaneously Fermented and Raw Food Products

**DOI:** 10.3390/foods14193395

**Published:** 2025-09-30

**Authors:** Thilakna Ampemohotti, Christopher Spooner, Sarah Eastwood, Aida Golneshin, Charles Brennan, Christopher Pillidge, Thi Thu Hao Van

**Affiliations:** 1 School of Science, RMIT University, Bundoora, VIC 3083, Australia; thilakna.ampemohotti@rmit.edu.au (T.A.); christopher.spooner@rmit.edu.au (C.S.); sarah.eastwood@rmit.edu.au (S.E.); charles.brennan@rmit.edu.au (C.B.); christopher.pillidge@rmit.edu.au (C.P.); 2 Edlyn Foods Pty Ltd., Epping, VIC 3076, Australia; agolneshin@edlyn.com.au

**Keywords:** fermented foods, oxidative stress, lactic acid bacteria (LAB), radical scavenging activity, hydrogen peroxide (H_2_O_2_), antioxidant mechanisms

## Abstract

An imbalance of pro-oxidants and antioxidants causes oxidative stress, contributing to various chronic diseases. Lactic acid bacteria (LAB) have recognised antioxidant activities that can help reduce oxidative stress. This study isolated fifty LAB strains from various fermented foods and raw vegetable products and evaluated their radical scavenging activity using DPPH and ABTS assays. Among them, four strains *Lacticaseibacillus paracasei* D2, *Lacticaseibacillus rhamnosus* JL, *Latilactobacillus sakei* F1, and *Weissella cibaria* JLK were selected and assessed for their tolerance to hydrogen peroxide (H_2_O_2_). Antioxidant mechanisms were investigated at the molecular level. Genome analysis revealed that the catalase gene (*katE*) was present in *L. sakei* F1, while it was absent in other strains. After exposure to H_2_O_2_, expression of genes associated with various antioxidant systems in the bacterial strains were measured at different growth phases. The results revealed that NADH oxidase-peroxidase, thioredoxin, and glutathione peroxidase systems play a role in antioxidant activity in *L. paracasei* D2 and *L. rhamnosus* JL strains, while genes associated with these systems in *L. sakei* F1 and *Weissella cibaria* JLK strains showed no upregulation. A different antioxidant mechanism was observed in *L. sakei* F1. The findings suggest that the four LAB strains are promising probiotic candidates with significant enzymatic or non-enzymatic antioxidant properties, which may aid in developing antioxidant-rich functional foods.

## 1. Introduction

Oxidative stress in health occurs when intracellular oxygen radical levels increase, leading to damage in lipids, proteins, and DNA [1,2,3,4]. An excessive accumulation of reactive oxygen species (ROS), such as superoxide anion (O_2_**^−^**), hydroxyl radicals (HO·), and hydrogen peroxide (H_2_O_2_), can lead to cellular damage, which may contribute to the development of chronic conditions such as arthritis, diabetes, neurodegenerative disorders, cardiovascular diseases, and cancer [5,6,7,8]. To cope, most living organisms have developed protective mechanisms, including enzymatic as well as non-enzymatic antioxidant mechanisms [9,10,11,12]. However, these defence systems are often insufficient alone to fully prevent oxidative damage. To counteract this, antioxidants in the diet (either natural or exogenous food additives) are able to protect the human body from oxidative damage. For example, vitamins C and E may be added to foods, while synthetic antioxidants such as butylated hydroxyanisole (BHA) and butylated hydroxytoluene (BHT) are widely used to delay lipid oxidation. However, some safety concerns have emerged regarding the latter due to potential liver toxicity and carcinogenic effects [13,14].

Humans are routinely exposed to lactic acid bacteria (LAB) and their metabolic byproducts in the diet, and they provide a wide range of health benefits, including supporting intestinal homeostasis, detoxifying harmful compounds, and strengthening immune responses [15,16,17]. In addition, certain LAB strains possess significant antioxidant properties, and these can be utilised as effective, natural antioxidants [9]. LAB are a broad group of Gram-positive bacteria commonly found in nature, and they exhibit inherent antioxidant activity for managing oxidative stress through both enzymatic and non-enzymatic mechanisms. The enzymatic mechanisms contain different systems, such as NADH oxidase-peroxidase [18], glutathione peroxidase [19], thioredoxin (Trx) [20,21], as well as catalase [22] and superoxide dismutase [23]. In addition, LAB can produce various non-enzymatic antioxidant metabolites such as glutathione (GSH), butyrate, and folate, and metal-binding proteins (MBPs), which efficiently lower ROS to non-toxic levels and thus shield cells from oxidative stress [24,25,26]. Moreover, there has been a growing interest in heat-killed (HK) LAB, as they can be stored longer and transported more easily than live LAB [27]. Although HK LAB do not secrete soluble metabolites, they may exert their effects through alternative mechanisms. However, there remains a lack of conclusive evidence regarding the impact of non-viable LAB on the biological activities of the host [28,29].

The oxygen sensitivity of LAB strains varies considerably, despite all being classified as aerotolerant anaerobes. These bacteria generally lack a functional electron transport chain, although they may contain some components that compromise their ability to survive in aerobic environments. High oxygen levels promote the formation of ROS. When these ROS accumulate, they induce oxidative stress, which ultimately causes cell death [6,30]. However, the antioxidant mechanisms of LAB are intricate, with different strains utilising distinct approaches. It has been proposed that LAB may exert antioxidant effects by scavenging ROS, chelating metals, increasing antioxidant enzyme levels, and modulating the gut microbiota [6]. A variety of methods have been developed to evaluate the antioxidant properties of LAB, including assays targeting free radical scavenging, metal ion chelation, enzyme activity, and antioxidant end-products. Due to the absence of a standardised testing protocol, it is difficult to directly compare the antioxidant capacities of different strains. Therefore, a combination of analytical approaches is necessary to effectively identify and characterise novel probiotic LAB strains for use in food production as functional food additives [31].

Earlier studies have primarily concentrated on single bacterial strains, exploring their molecular mechanisms without offering comparisons across different strains. Although *Weisella cibaria* is gaining recognition for its potential probiotic applications in humans, research on its antioxidant activity remains limited. In addition, there has been little investigation into the molecular mechanisms of *W. cibaria* and its gene expression in response to oxidative stress. Understanding the role of environmental LAB in antioxidant processes may provide new insights into how microorganisms influence both food production and human health. Employing specific bacterial strains could potentially reduce the use of synthetic antioxidants in foods by leveraging the strain’s natural antioxidant properties. This study reports on the isolation of 50 LAB strains from raw and fermented food products, evaluates their in vitro antioxidant properties, and investigates the antioxidant mechanisms of four selected strains at the genetic level to investigate their potential as natural beneficial strains for food applications.

## 2. Materials and Methods

### 2.1. Isolation of LAB Strains

LAB strains were isolated from seven types of raw vegetables and fermented food products. The dilution-plate method was used to isolate LAB on de Man, Rogosa, and Sharpe (MRS) agar and MRS broth (BD Biosciences, Sparks, MD, USA). A 10 g food sample was mixed with 40 mL of sterilised MRS broth to create a 20% suspension. Decimal dilutions ranging from 10^−1^ to 10^−10^ were prepared to reduce microbial concentration, and 100 μL of each dilution was plated onto MRS agar plates. The plates were incubated at 37 °C for 24–48 h in anaerobic conditions generated by AnaeroGen™ sachets (Oxoid, Thermo Fisher Scientific, Basingstoke, UK). Following incubation, colonies exhibiting different morphological characteristics were selected and sub-cultured twice to ensure purity for further analysis [32,33]. For long-term preservation, the stock bacterial cultures in MRS broth containing 30% (*v*/*v*) sterile glycerol (Sigma-Aldrich, St. Louis, MO, USA) were stored at −80 °C [33].

### 2.2. Preparation and Identification of Isolates for Matrix-Assisted Laser Desorption/Ionization Time-of-Flight Mass Spectrometry (MALDI-TOF MS)

Frozen stock cultures were revived for 24 h at 37 °C in freshly prepared MRS broth. Each culture was streaked onto MRS agar plates to obtain single colonies and incubated at 37 °C for 12–18 h [34]. Colonies were applied onto the MALDI-TOF MS spots, after which 1 μL of formic acid (70%, *v*/*v*) (Sigma-Aldrich, St. Louis, MO, USA) was added and the samples were allowed to dry at room temperature. Dried spots were covered with 1 μL of α-cyano-4-hydroxycinnamic acid (HCCA) matrix (Bruker Daltonics, Bremen, Germany) prepared from Bruker’s instant soluble formulation. Then the samples were left to dry at room temperature [35]. Bacterial isolates were identified using a Bruker MALDI Biotyper system (Bruker Daltonics, Bremen, Germany). Data were analysed using the Bruker MALDI Biotyper 4.1.80 software package.

### 2.3. Preparing Live and HK LAB for the Antioxidant Assays

The strains were cultured in MRS broth at 37 °C for 16 h. HK LAB samples were prepared by heating the cells at 80 °C for 30 min. After the heat treatment, bacterial suspensions were serially diluted in MRS broth up to 10^−5^ and spread onto MRS agar plates. The plates were then incubated at 37 °C for 48 h to observe colony growth and confirm the complete loss of bacterial viability. Both live and heat-killed (HK) cells were subsequently centrifuged using the Shandon Cytospin^®^ 4 cytocentrifuge (Thermo Fisher Scientific, Waltham, MA, USA) at 14,000× *g* for 5 min and washed twice with 0.85% (*w*/*v*) saline solution. The bacterial cells were subsequently resuspended in saline water and diluted to a final concentration of 10^7^ CFU/mL [27,36].

### 2.4. Ability to Scavenge DPPH Free Radical and ABTS Radicals

In vitro antioxidant activities of isolates were assessed based on their scavenging ability against 1,1-diphenyl-2-picryl-hydrazyl (DPPH·) and 2,2’-azino-bis (3-ethylbenzothiazoline-6-sulfonic acid (ABTS·) (Sigma-Aldrich, St. Louis, MO, USA). The ABTS radical scavenging activity was assessed following the method described by Shi et al. [37] with slight modifications. Briefly, the ABTS radical cation was generated by mixing 7 mM ABTS with 2.45 mM potassium persulfate (1:1 *v*/*v*) and allowing the mixture to incubate in the dark at room temperature for 16–18 h. The resulting ABTS solution was then diluted with methanol until an initial absorbance of 0.700 ± 0.020 at 734 nm. For the assay, 50 µL of live or HK sample was mixed with 100 µL of ABTS solution and incubated at room temperature for 10 min. The absorbance of the reaction mixture was then measured at 734 nm. Each assay was conducted in triplicate, and the scavenging rate was determined using the formula:

ABTS scavenging activity (%) = (1 − A test/A control) × 100%

where: A test = absorbance of the test sample; A control = absorbance of the saline water control sample.

DPPH radical scavenging activity was assessed according to the method described by Lin and Yen [38] with some modifications. DPPH solution was prepared freshly by dissolving DPPH in absolute ethanol to a final concentration of 0.2 mM. For the assay, 80 µL of live or HK cells were combined with 100 µL of DPPH solution and incubated in the dark for 30 min. For the control, the bacterial suspension was replaced with saline water. Meanwhile, the blank group was set up by substituting the DPPH radicals with an equal volume of ethanol. The absorbance was recorded at 517 nm. The scavenging activity was calculated using the formula:

DPPH scavenging activity (%) = [1 − (A sample − A blank)/A control] × 100

### 2.5. Growth of the Strains in the Presence of H_2_O_2_

H_2_O_2_ tolerance was examined as described in previous studies [39,40] with some changes. The eight top-performing strains common across both antioxidant assays were further evaluated for their tolerance to oxidative stress by culturing them in MRS broth supplemented with increasing concentrations of H_2_O_2_ up to 0–6 mM for 24 h at 37 °C. Tolerance was assessed by visually observing the turbidity of cells and checking the mass of bacterial pellets after centrifugation. Based on the results of DPPH, ABTS antioxidant assays and H_2_O_2_ tolerance, four bacterial strains were selected for further study.

### 2.6. Whole Genome Sequencing and Detection of Genes Involved in Antioxidant Activity

Pure cultures of *Lacticaseibacillus paracasei* D2, *Lacticaseibacillus rhamnosus* JL, *Latilactobacillus sakei* F1 and *Weissella cibaria* JLK were grown in MRS broth up to 10^8^ CFU/mL, and DNA was extracted using the DNeasy^®^ PowerSoil^®^ Kit (Qiagen, Hilden, Germany) as per the manufacturer’s instructions. Total DNA quality and concentrations were measured using NanoDrop and QubitTM Fluorometric Quantitation (ThermoFisher, Waltham, MA, USA), respectively. Whole genome sequencing was carried out using Illumina Nextera XT DNA Library Preparation Kit and sequenced on an Illumina MiSeq platform with 2 × 300 bp reads. The genomes were assembled using A5-miseq [41], and the assembled genomes were annotated with the Rapid Annotation using Subsystem Technology pipeline (RAST) [42]. Genes associated with antioxidants were identified in the RAST output and also confirmed with Basic Local Alignment Search Tool (BLAST+ 2.17.0) search [43].

### 2.7. Bio-Screen Assay and Expression Levels of Genes Involved in Antioxidant Activity

The bio screen assay was conducted with the four most promising LAB strains based on DPPH, ABTS antioxidant results and the tolerance of H_2_O_2_ to assess biomass proliferation. Pure cultures of *L. sakei* F1, *L. paracasei* D2, *L. rhamnosus* JL, and *W. cibaria* JLK were standardised to 10^7^ CFU/mL and inoculated into MRS broth containing varying concentrations of H_2_O_2_ from 0.5–8 mM. The control medium for all strains contained no added H_2_O_2_. Then, the optical density at 600 nm (OD_600_) was recorded every 3 h for 40 h after brief shaking of the plate for strains JL, JLK, and D2 [44]. For strain F1, OD_600_ measurements were taken at specific time points: 0, 4, 8, 10, 13, 23, 26, and 29 h with high concentrations of H_2_O_2_ (0–8 mM) due to the shown higher tolerance to H_2_O_2_. For all strains, cells were collected at mid-logarithmic phase and the start of the stationary phase for RNA extraction, except strain F1, where cells were harvested at the start of the stationary phase only [44]. The libraries were prepared using the Illumina TruSeq Stranded Total RNA with Ribo-Zero Plus workflow (rRNA depletion) (Illumina, Inc., San Diego, CA, USA), and sequencing was carried out with the Illumina NovaSeq 6000 platform (Illumina, Inc., San Diego, CA, USA), and 150-bp paired-end reads were generated at the Australian Genome Research Facility (AGRF). RNA-seq data, including quality check, mapping reads, and quantification of gene expression levels, were analysed using the Galaxy Australia platform (https://usegalaxy.org.au, accessed on 23 May 2025).

### 2.8. Statistical Analysis

Statistical analyses were conducted using Minitab^®^ 18 and GraphPad Prism 10.4.2 statistical software. Results are expressed as mean ± standard error (SEM), with all experiments performed in triplicate. The mean values and standard errors were calculated, and a probability level of *p* < 0.05 was utilised to test the statistical significance of the experimental data. During the screening phase, fifty bacterial strains were evaluated using a Two-way Analysis of Variance (two-way ANOVA) in Minitab^®^ 18. To assess the significance of differences between group means in multiple comparisons, Tukey’s post-hoc test was applied, enabling the identification of the top strains with the highest antioxidant activities. For the selected top eight strains, a two-way ANOVA followed by Šídák’s multiple comparisons test was conducted using GraphPad Prism 10.4.2 statistical software to uncover treatment-specific differences among the strains.

## 3. Results

### 3.1. Identification of LAB Using MALDI-TOF Analysis

For species-level identification, LAB strains were subjected to MALDI-TOF analysis (Table 1). A total of 50 isolates were identified, with the majority belonging to the *Latilactobacillus* genus, including *L. sakei* (12 isolates) and *L. curvatus* (8). Other genera included *Leuconostoc: L. mesenteroides* (5), *L. citreum* (3)*; Lacticaseibacillus: L. paracasei* (5), and *L. rhamnosus* (1); *Weisella: W. cibaria* (3) and *W. koreensis* (1); *Levilactobacillus brevis* (3); *Pediococcus: P. pentosaceus* (2) and *P. acidilactici* (1); *Lactococcus lactis* (2); *Lactiplantibacillus plantarum* (2); *Furfurilactobacillus rossiae* (1); *Lactobacillus delbrueckii* (1).

### 3.2. DPPH and ABTS Free Radical Scavenging Activities

**Figure 1:**
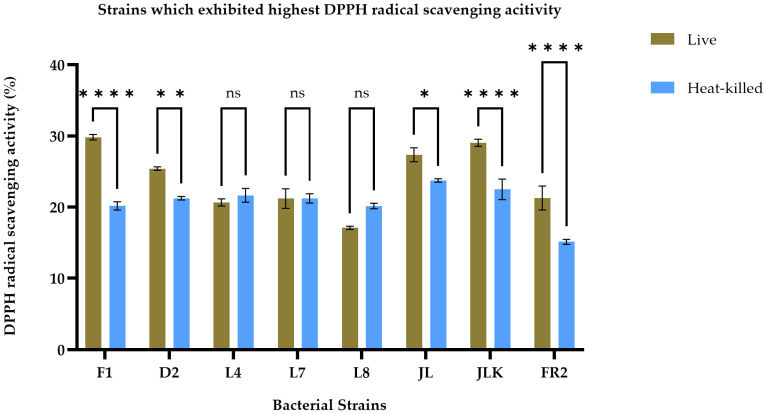
DPPH radical scavenging activity of live (brown bars) and heat-killed (blue bars) bacterial strains. Statistical analysis by two-way ANOVA followed by Šídák’s multiple comparisons test revealed strain-dependent differences. Strains F1, D2, JL, JKL and FR2 exhibited significantly higher scavenging activity in live cells compared to their heat-killed counterparts (*p* < 0.01 to *p* < 0.0001). No significant differences were observed for strains L4, L7, and L8 (*p* > 0.05). Data are presented as mean ± SEM (*n* = 3). ns = not significant; * *p* ≤ 0.05, ** *p* ≤ 0.01, **** *p* ≤ 0.0001.

**Figure 2:**
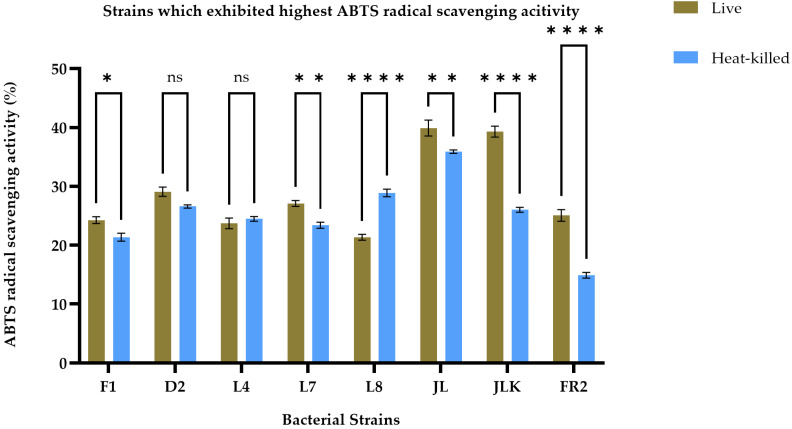
ABTS radical scavenging activity of live (olive brown bars) and heat-killed (blue bars) bacterial strains. Statistical analysis by two-way ANOVA followed by Šídák’s multiple comparisons test revealed treatment-specific differences across strains. L8 showed a modest but significant increase in heat-killed cells compared with live forms. No significant differences were observed for D2 and L4. Strains F1, L7, JL, JLK, and FR2 exhibited significantly higher activity in live cells than in their heat-killed counterparts (*p* < 0.01 to *p* < 0.0001). Data are presented as mean ± SEM (*n* = 3). ns = not significant; * *p* ≤ 0.05, ** *p* ≤ 0.01, **** *p* ≤ 0.0001.

The antioxidant activity of live and HK LAB strains, isolated from both raw and fermented food products, was assessed by measuring their ability to scavenge free radicals using the ABTS and DPPH assays, as presented in Table 2. The findings indicate that both live and HK LAB strains have varying abilities to scavenge different free radicals. After conducting a screening process, we identified the top eight strains, including F1, D2, L4, L7, L8, JL, JLK, and FR2, based on their consistently high antioxidant activity across two assays, as illustrated in Figure 1 and Figure 2. In Figure 1, the F1 strain demonstrated the highest DPPH radical scavenging activity in live cells, recorded at 29.84 ± 0.39%, while the JL strain exhibited the highest activity in its HK form, measuring 23.75 ± 0.24%. Overall, live cells demonstrated a significantly (*p* < 0.05) strong DPPH radical scavenging ability across all strains, except for L4, L7, and L8, which did not exhibit significant differences between the live and HK groups (*p* < 0.05). Results from the ABTS radical scavenging activity tests indicated that the JL strain exhibited the highest radical scavenging activity in both live and HK forms, showing values of 39.90 ± 1.34% and 35.87 ± 0.29%, respectively. Generally, live bacterial cultures demonstrated significantly higher radical scavenging activity (*p* < 0.05), except for the L8 strain. Furthermore, there were no significant differences between the two groups for the D2 and L4 strains. The findings indicate that live cultures typically demonstrate superior antioxidant activity; however, HK forms of certain strains also reveal noteworthy antioxidant properties, which necessitated further investigation.

### 3.3. Genome Sequences of L. paracasei D2, L. rhamnosus JL, W. cibaria JLK, L. sakei F1

After the assessment of antioxidant activity, the oxidative stress tolerance of selected strains was evaluated in the presence of H_2_O_2_. Based on the ability of strains to grow under different H_2_O_2_ conditions, four strains, including F1, JLK, JLFS, and D2, were selected for further characterisation with the bio-screen assay and assessed for the expression levels of genes involved in antioxidant activity.

The genome size and GC content of the four bacterial strains are shown in Table 3. The genome size ranges from 1.98 to 3.36 Mbp. Among them, strain F1 exhibited the smallest genome size, while D2 had the largest genome. The GC content also showed considerable variation across the strains ranging from 41 to 46.6%.

Based on the annotation results and a literature search on common antioxidant-related genes in LAB [44,45,46], a total of eight antioxidant-related genes were identified in the strains, as shown in Table 4. *L. sakei* F1 is the only strain that contains the *katE* gene, and *L. rhamnosus* JL is the only strain with the *nox* gene.

### 3.4. Analysis of the Transcript Levels of the Genes Involved in Antioxidant Activity in LAB Strains Exposed to H_2_O_2_

**Figure 3:**
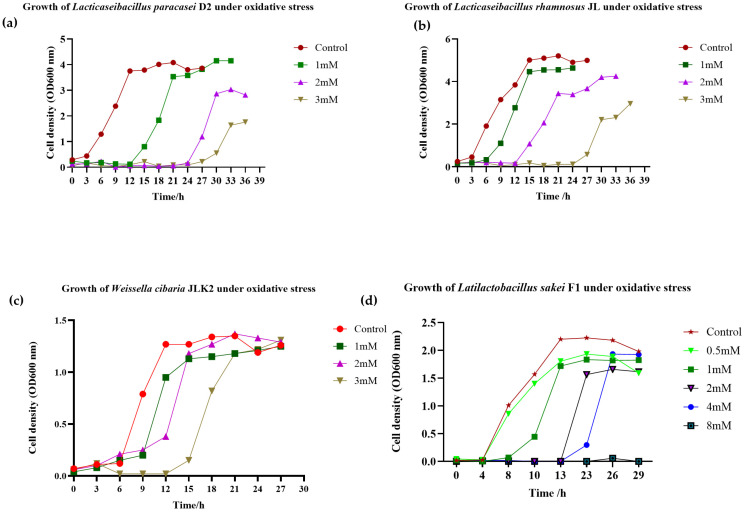
Growth of *Lacticaseibacillus paracasei* D2 (**a**), *Lacticaseibacillus rhamnosus* JL (**b**), and *Weissella cibaria* JLK (**c**) under oxidative stress. The strains were cultured in MRS broth supplemented with H_2_O_2_ at concentrations of 1, 2, and 3 mM. In contrast, *Latilactobacillus sakei* F1 (**d**) was grown under a broader range of H_2_O_2_ concentrations (0.5, 1, 2, 4, and 8 mM).

The growth of JLK, JL and D2 showed tolerance against H_2_O_2_ at 1, 2, and 3 mM as illustrated in Figure 3a–c. F1 exhibited bacterial growth under 4 mM, as shown in Figure 3d. The presence of H_2_O_2_ reduced the bacterial growth, and the lag phases were considerably prolonged with the increasing H_2_O_2_ concentrations, indicating that H_2_O_2_ caused oxidative damage to the bacteria.

To unravel the potential mechanism of antioxidant activity in selected strains, gene expression via RNA was measured. We focused on the upregulation of antioxidant-related genes at two growth phases: the mid-log and the start of the stationary phases. This was conducted across two concentrations per strain: control and 2 mM for strain D2, control and 2 mM for strain JL, and control and 3 mM for strain JLK. For strain F1, the relative expression levels were assessed at the start of the stationary phase using three different concentrations: control, 2 mM and 4 mM.

As shown in Table 5, genes associated with the NADH oxidase-peroxidase system, including *nox* and *npx*, showed considerable upregulation in D2 and JL strains. Specifically, the *npx* gene exhibited a notable upregulation at the mid-log phase, with increases of 1.42-fold in D2 and 1.44-fold in JL, suggesting their direct involvement in antioxidant activity. The *nox* gene, which is absent in the D2 strain but present in the JL strain, also demonstrated upregulation at the mid-log phase. Furthermore, genes associated with the Trx systems, such as *tpx* and *trxB*, showed mild upregulation in both strains. The glutathione peroxidase system, including *gpx* and *gshR* genes, displayed slight upregulation in the early stationary phase in the D2 strain. In contrast, these genes showed a slight upregulation at the mid-log phase in the JL strain. All isolates contain the *sodA* gene, but none of the strains exhibited any upregulation for this gene, indicating that *sodA* might not directly contribute to antioxidant activity in these strains. For the *L. sakei* F1 and *W. cibaria* JLK strains, only *katE* and *npx* showed upregulation at 4 mM and 3 mM H_2_O_2_, with fold changes of 1.26 and 1.10, respectively. Interestingly, for the F1 strain only, significant upregulation of dihydroorotate dehydrogenase was observed (5.45—fold change). This enzyme does not directly neutralise ROS but indirectly contributes to antioxidant activity [47].

## 4. Discussion

Living organisms continuously produce ROS during their physiological activities. Malfunctioning oxidative systems and an increase in free radicals leads to oxidative stress, which can irreversibly damage cells and their components [48]. LAB have been extensively studied for their antioxidant activity using various approaches. Their antioxidant effects have been demonstrated, including free radical-scavenging capacities, lipid peroxidation-inhibition capacities, and metal-chelating abilities, along with a variety of antioxidant enzyme activities [28,36,49]. Thermal treatment or heat-killing (HK) is the most common method for microbial inactivation of live probiotic strains to produce ‘paraprobiotics’ [28]. Research has shown that LAB subjected to thermal treatment exhibit a wide range of biological activities, including some with beneficial physiological effects [28,50,51,52]. In this study, fifty live and HK LAB strains, isolated from fermented and raw food products, were tested for their in vitro antioxidant properties based on biochemical assays of DPPH- and ABTS-scavenging activities. Notably, the results from the ABTS radical cation scavenging assay were comparable to those from the DPPH radical scavenging tests, showing similar trends in the mean values across most bacterial strains with varied treatments. Both antioxidant assays can be monitored with a spectrophotometer. Specifically, the ABTS assay is based on the formation of blue/green ABTS·+, which can be reduced by antioxidants, while the DPPH assay relies on the reduction of purple DPPH· to its non-radical hydrazine form, leading to a loss of colour [53]. In addition to these assays, there are other methods for assessing the antioxidant activity of LAB, such as the ferric reducing antioxidant power (FRAP) assay, the hydroxyl radical scavenging test, the ferrous ion-chelating assay, and the determination of superoxide anion radical scavenging capacity [54]. In the present study, we considered that a bio-screen growth assay under H_2_O_2_ stress and transcriptomic analysis of antioxidant-related genes, in addition to the two chemical assays, produced more complete information. This approach offered a multifaceted evaluation of the antioxidant activity of the strains by providing both physiological and molecular evidence of antioxidant capacity.

Overall, most of the live LAB strains exhibited higher antioxidant potential in both the DPPH and ABTS assays compared to HK cells. These results are in line with the previous work done by Li, et al. [55]. They investigated the radical scavenging activity of live/HK *L. sakei* MS103 and found that live cells exhibited higher antioxidant activity. This could be attributed to certain cell surface-active compounds, such as proteins, polysaccharides, exopolysaccharides (EPS), and lipoteichoic acid, which have been observed in *L. plantarum* C88, *Bifidobacterium* spp., and *L. brevis* KU15147, as documented by Li [56], Yi, et al. [57], Kim, et al. [58]. It is interesting to note that HK L8 demonstrated higher antioxidant activity compared to its live cells in the ABTS assay. However, in the DPPH assay, both L8 live cells and HK cells exhibited no significant differences. We observed similar results in a study conducted by Xu, et al. [59] with *Loigolactobacillus coryniformis* NA-3, where the heat-killed strain also exhibited activities similar to those of live bacteria. Additionally, in our study, the HK forms of certain strains, including SK1, D6, SK 5-1, RSK 1, SK^®^3 4, Broc 03, Kim 1-1, Kim 1-2, and Kim 2-1, demonstrated significantly higher radical scavenging activity (*p* < 0.05) compared to their live forms in both screening assays. The relationship between the HK LAB and bioactivity remains unclear. Some studies have shown that excessive heating at 121 °C can significantly reduce the antioxidant and anti-inflammatory activity of probiotics. This reduction may be attributed to a decrease in the content of metabolites that possess these beneficial activities [60].

Following screening using these biochemical assays, four strains were selected for their maximum antioxidant potential: *Lacticaseibacillus paracasei* D2, *Lacticaseibacillus rhamnosus* JL, *Latilactobacillus sakei* F1, and *Weissella cibaria* JLK. Another method was used for evaluating oxidative stress tolerance is to assess cell viability in the presence of H_2_O_2_. H_2_O_2_ is considered a mild oxidant; however, it can permeate the cell membrane and generate more ROS and initiate oxidative damage [9]. Lactobacilli are known to generate O_2_^-^ and H_2_O_2_ and accumulate millimolar concentrations of peroxide. Mechanisms to counteract the toxicity of ROS are crucial for survival in oxygen-rich environments. To prevent oxidative stress, all bacteria, including LAB, possess enzymatic and non-enzymatic defensive mechanisms [24]. Enzymatic mechanisms typically involve superoxide dismutase (SOD) and hydrogen peroxidase enzymes such as catalases (CAT) and peroxidases which serve as the primary defence against O_2_^−^ and H_2_O_2_, respectively [61]. Specifically, SOD catalyses the conversion of superoxide anions to oxygen, helping to prevent H_2_O_2_-induced cell damage. CAT is the enzyme that catalyses the decomposition of H_2_O_2_, playing a vital role in protecting cells from oxidative damage caused by ROS. LAB generally lack CAT because they cannot synthesise heme [62]. An, et al. [63] reported *that L. rhamnosus* is deficient in *katE* and *sodA/sodB* genes, which matches our findings for the absence of these genes in *L. rhamnosus* JL. Additionally, we observed that *W. cibaria* JLK also lacks both *katE* and *sodA/sodB* genes. Although we found that *L. paracasei* D2 and *L. sakei* F1 contain the *sodA* gene, our findings did not support its active involvement in antioxidant properties, despite its well-documented role in this regard for other bacteria. We observed the downregulation of *sodA* in both strains across all concentrations and growth phases, suggesting that *sodA* does not contribute to the antioxidative properties of the tested strains. This lack of involvement may be due to strain-specific differences. Additionally, *L. sakei* F1 is the only strain that contains the *katE* gene, which exhibited upregulation in the presence of H_2_O_2_. *L. sakei* F1 shared other common antioxidant-related genes, including *npx*, *tpx*, *trxB*, *gshR*, *sodA*, as well as *katE*, in agreement with the study by Li, et al. [55]. They found that *L. sakei* MS103, isolated from pickled garlic, exhibited antioxidant activity and stress response in the presence of H_2_O_2_, with upregulated expression of the *gshR2*, *gshR4*, *gpx*, and *npx* genes. However, in our study, these genes did not show active involvement in the presence of H_2_O_2_, except for *katE*. Nevertheless, significant upregulation occurred in dihydroorotate dehydrogenase (5.45- fold change) at 4 mM H_2_O_2_ exposure in the F1 strain, an enzyme that does not directly neutralise ROS but indirectly contributes to antioxidant activity through influencing cellular redox balance [47].

The glutathione peroxidase system, including *gshR* and *gpx* genes, is another system that plays a crucial role in protecting against oxidative damage by oxidising glutathione disulphide, thereby reducing H_2_O_2_ levels [64]. Furthermore, the glutathione peroxidase system is involved in redox regulation [65,66]. The relationship between H_2_O_2_-induced oxidative stress and the glutathione peroxidase system in Lactobacilli has been rarely studied. However, in this study, we observed that the expression levels of *gpx* and *gshR* increased at the beginning of the stationary phase in *L. paracasei* D2 strain. This suggests that these enzymes also play a role in the antioxidant activity of *L. paracasei* D2 by regulating oxidative stress. Recent studies have reported that *L. paracasei* has promising antioxidant effects [67,68]. The strain *L. paracasei* TDM-2, isolated from a fermented dairy product, demonstrated high antioxidant capacity and tolerance against 2 mM H_2_O_2_ [69]. In addition, Yang, et al. [46] found that *L. paracasei* strain M11-4 isolated from fermented rice has antioxidant potential and survives under 2.5 mM H_2_O_2_. It upregulated the expression of its antioxidant enzyme systems, notably the Trx and glutathione peroxidase antioxidant pathways. The Trx system is known to respond to H_2_O_2_-induced oxidative stress in lactobacilli. In agreement with this finding, we observed elevated expression of genes associated with Trx and glutathione peroxidase pathways, indicating that both play an important role in the antioxidant activity of *L. paracasei* D2.

The gene *npx* is a well-known gene harboured by microorganisms that are involved in the scavenging of H_2_O_2_ [70]. It directly reduces H_2_O_2_ to water using NADH [55]. Strains without *katE* rely on *npx* and high intracellular manganese for peroxide scavenging [63]. In this study, we observed that *npx* appeared to play a role in the antioxidant activity of strain *L. paracasei* D2 and *L. rhamnosus* JL during the mid-log phase. Additionally, *L. rhamnosus* strains were found to possess *nox* and *npx* genes, possibly managing ROS, and often accumulate high levels of manganese, which can non-enzymatically suppress superoxides [31]. Our findings align with this prior research, as *nox* and *npx* were noticeably upregulated in the mid-log phase. Moreover, the results revealed that most antioxidant-related genes are growth phase-dependent, supporting the findings of Wu, et al. [71], who investigated the antioxidant mechanisms of *L. plantarum* ZJ316. The strain exhibited upregulation of the *nox* and *npx* genes by 1.72-fold and 1.41-fold, respectively, during the stationary phase under 2.5 mM H_2_O_2_ stress.

There is a limited number of studies on the antioxidant mechanisms of the *W. cibaria* species. Yu, et al. [72] showed that the strain *W. cibaria* JW15, isolated from kimchi, exhibited considerably higher free radical scavenging activity than other strains. It efficiently neutralised DPPH, ABTS, and hydroxyl radicals, and also inhibited linoleic acid peroxidation, indicating that JW15 can both prevent lipid oxidation and directly scavenge ROS [72]. Our study found that all common antioxidant-related genes in *W. cibaria* JLK were not upregulated under H_2_O_2_ stress, except for *npx*, which showed slight upregulation. The classical *katE* or *sodA* are also absent in this strain, further demonstrating that its antioxidant activity is likely to rely on non-enzymatic factors such as EPS and antioxidant metabolites [24].

## 5. Conclusions

In this study, four LAB strains isolated from fermented foods or raw vegetables exhibiting strong in vitro antioxidant activity were identified through a series of experimental evaluations. *L. paracasei* D2, *L. rhamnosus* JL, *L. sakei* F1 and *W. cibaria* JLK demonstrated notable tolerance to H_2_O_2_ stress and effective scavenging activity against free radicals. The three different antioxidant systems, including NADH oxidase-peroxidase, thioredoxin, and glutathione peroxidase systems, appear to play an important role in the antioxidant activity of strains D2 and JL. In contrast, the role of these systems in the antioxidant activities of strains JLK and F1 was not obvious, with only *npx* found to be upregulated under H_2_O_2_ stress in strain JLK. Instead, non-enzymatic antioxidant factors and indirect antioxidant pathways may be involved, as demonstrated by the significant upregulation of dihydroorotate dehydrogenase in strain F1. The catalase pathway also seems to be involved in the antioxidant activity of this strain. These findings offer new insights into the molecular mechanisms underlying the antioxidant activity of LAB strains. Further studies are needed to fully elucidate the antioxidant pathways involved. Based on their antioxidative properties, these four LAB strains have promising potential as probiotic candidates for the development of functional foods with superior antioxidant benefits.

## Figures and Tables

**Table 1:** Isolated microorganisms from different food products.

Isolated Food Sample	Organism ID	Species Name
Blue Cheese	F3	*Lactococcus lactis*
Homemade sauerkraut	L4	*Lacticaseibacillus paracasei*
	L5	*Lactiplantibacillus plantarum*
	L6	*Levilactobacillus brevis*
	L7	*Latilactobacillus curvatus*
	L8	*Pediococcus pentosaceus*
	SK1	*Leuconostoc citreum*
	SK21	*Latilactobacillus curvatus*
	D2	*Lacticaseibacillus paracasei*
	D1	*Pediococcus pentosaceus*
	D6	*Lactococcus lactis*
	SK 5-1	*Levilactobacillus brevis*
	SK 5-2	*Furfurilactobacillus rossiae*
	SK 5-3	*Lacticaseibacillus paracasei*
	RSK 1	*Lacticaseibacillus paracasei*
	RSK 2	*Levilactobacillus brevis*
	SKR 32	*Lactiplantibacillus plantarum*
	SK ^®^ 3 4	*Lacticaseibacillus paracasei*
	SK 10-5	*Leuconostoc mesenteroides*
Raw cabbage	Cab 01	*Leuconostoc citreum*
Raw broccoli	Broc 03	*Weissella cibaria*
	RB2	*Leuconostoc citreum*
Homemade Kimchi	F1	*Latilactobacillus sakei*
	F2	*Latilactobacillus curvatus*
	JL	*Lacticaseibacillus rhamnosus*
	T	*Latilactobacillus sakei*
	JLK	*Weissella cibaria*
	DT3	*Latilactobacillus sakei*
	KM 5	*Pediococcus acidilactici*
	Kim 1-1	*Latilactobacillus sakei*
	Kim 1-2	*Latilactobacillus curvatus*
	Kim 2-1	*Latilactobacillus sakei*
	Kim 2-4	*Latilactobacillus curvatus*
	Kim 3-1	*Latilactobacillus sakei*
	Kim 3-2	*Weissella koreensis*
	Kim 4-1	*Latilactobacillus sakei*
	Kim 4-2	*Latilactobacillus curvatus*
	Kim 4-4	*Leuconostoc mesenteroides*
	Kim 5-1	*Latilactobacillus curvatus*
	Kim 5-4	*Leuconostoc mesenteroides*
	Kim 6-1	*Latilactobacillus sakei*
	Kim 7-1	*Latilactobacillus sakei*
	Kim 8-3	*Leuconostoc mesenteroides*
	Kim 8-5	*Latilactobacillus sakei*
	Kim 9-7	*Latilactobacillus sakei*
	Kim 9-8	*Latilactobacillus curvatus*
Fermented radish	FR 1	*Latilactobacillus sakei*
	FR 2	*Leuconostoc mesenteroides*
Kombucha	HA	*Weissella cibaria*
	HC	*Lactobacillus delbrueckii*

**Table 2:** Antioxidant properties of LAB strains.

Strains	ABTS (%)		DPPH (%)	
	Live	Heat-Killed	Live	Heat-Killed
F1	24.24 ± 0.59 ^F−J^	21.36 ± 0.68 ^J K^	29.84 ± 0.39 ^A^	20.18 ± 0.58 ^E–I^
F2	25.76 ± 0.34 ^D–H^	22.93 ± 0.40 ^H–K^	15.12 ± 0.35 ^J K L^	12.56 ± 0.46 ^L–O^
F3	13.58 ± 0.76 ^N–T^	16.99 ± 0.40 ^L M N^	10.92 ± 0.43 ^M–R^	21.85 ± 0.10 ^D E F^
D2	29.08 ± 0.78 ^D^	26.59 ± 0.27 ^D –G^	25.42 ± 0.24 ^B C D^	21.25 ± 0.24 ^E–H^
L4	23.70 ± 0.90 ^F–J^	24.46 ± 0.41 ^F–J^	20.67 ± 0.51 ^E–I^	21.67 ± 0.96 ^D–G^
L5	12.05 ± 0.42 ^Q–W^	14.16 ± 0.43 ^N–R^	13.69 ± 0.25 ^K–N^	13.02 ± 0.18 ^L–O^
L6	13.55 ± 0.32 ^N–T^	10.65 ± 0.29 ^S–W^	11.73 ± 0.43 ^L–Q^	7.57 ± 0.52 ^R–W^
L7	27.09 ± 0.51 ^D E F^	23.38 ± 0.52 ^G–J^	21.21 ± 1.37 ^E–H^	21.22 ± 0.65 ^E–H^
L8	21.33 ± 0.50 ^J K^	28.87 ± 0.63 ^D E^	17.08 ± 0.24 ^I J K^	20.16 ± 0.39 ^E–I^
SK1	12.92 ± 0.45 ^O–V^	23.42 ± 0.49 ^G–J^	12.00 ± 0.95 ^L–P^	17.92 ± 0.24 ^G–J^
SK21	4.60 ± 0.50 ^AB–AG^	10.33 ± 0.50 ^S–X^	2.95 ± 0.29 ^Y–AB^	7.86 ± 0.35 ^Q–V^
D1	22.15 ± 0.69 ^I J K^	17.80 ± 0.50 ^L M^	17.59 ± 0.89 ^H I J^	15.30 ± 0.29 ^J K L^
D6	5.90 ± 0.36 ^AA–AE^	14.91 ± 0.50 ^M–Q^	3.91 ± 0.58 ^W–AA^	17.04 ± 0.24 ^I J K^
SK 5-1	3.73 ± 0.52 ^AB–AG^	13.97 ± 0.58 ^N–S^	0.00 ± 0.00 ^AB^	12.29 ± 0.32 ^L–P^
SK 5-2	15.81 ± 0.61 ^M–P^	9.80 ± 0.14 ^V–Z^	9.49 ± 0.96 ^O–S^	4.17 ± 0.48 ^V–AA^
SK 5-3	13.49 ± 1.28 ^O–T^	4.62 ± 0.59 ^AB–AG^	14.51 ± 0.93 ^J–M^	0.00 ± 0.00 ^AB^
RSK 1	2.48 ± 0.31 ^AE–AH^	10.00 ± 0.33 ^T–Y^	5.42 ± 0.72 ^T–Z^	10.92 ± 0.43 ^M–R^
RSK 2	0.00 ± 0.00 ^AH AI^	2.18 ± 0.31 ^AF–AH^	0.00 ± 0.00 ^AB^	0.00 ± 0.00 ^AB^
SKR 32	12.07 ± 0.23 ^Q–W^	10.57 ± 0.32 ^T–W^	11.72 ± 1.22 ^L–Q^	12.50 ± 1.44 ^L–O^
SK ^®^ 3 4	1.99 ± 0.45 ^AG AH^	13.64 ± 0.38 ^N–T^	0.00 ± 0.00 ^AB^	10.34 ± 0.73 ^N–S^
SK 10-5	16.20 ± 0.31 ^M N O^	4.98 ± 0.57 ^AB–AG^	30.18 ± 0.64 ^A^	4.60 ± 1.19 ^U–AA^
Cab 01	4.23 ± 0.18 ^AB–AG^	12.18 ± 0.51 ^Q–V^	4.19 ± 0.46 ^V–AA^	9.67 ± 0.28 ^O–S^
Broc 03	6.66 ± 0.49 ^Y–AC^	14.33 ± 0.31 ^N–R^	0.00 ± 0.00 ^AB^	22.91 ± 1.05 ^D E F^
RB2	34.90 ± 0.24 ^c^	25.56 ± 0.31 ^E–I^	30.72 ± 0.89 ^A^	19.26 ± 1.18 ^F–I^
JL	39.90 ± 1.34 ^A^	35.87 ± 0.29 ^B C^	27.37 ± 0.97 ^A B C^	23.75 ± 0.24 ^C D E^
T	5.48 ± 0.55 ^AA–AF^	6.20 ± 0.58 ^AA–AD^	5.45 ± 0.70 ^T–Z^	7.92 ± 0.24^Q–V^
JLK	39.27 ± 0.93 ^A B^	26.02 ± 0.41 ^D–H^	29.05 ± 0.49 ^A B^	22.50 ± 1.44 ^D E F^
DT3	9.83 ± 0.74 ^V–Y^	0.00 ± 0.00 ^AH AI^	6.70 ± 0.48 ^S–Y^	0.00 ± 0.00 ^D E F^
KM 5	5.87 ± 0.25 ^AA–AE^	6.35 ± 0.19 ^Z–AD^	4.26 ± 0.34 ^U–AA^	5.26 ± 0.39 ^T–Z^
Kim 1-1	8.69 ± 1.27 ^W–AA^	11.33 ± 0.45 ^R–W^	5.48 ± 0.37 ^T–Z^	10.28 ± 0.35 ^N–S^
Kim 1-2	5.95 ± 0.17 ^AA–AD^	14.94 ± 0.55 ^M–Q^	7.50 ± 0.48 ^R–X^	21.02 ± 0.15 ^C D E^
Kim 2-1	5.57 ± 0.47 ^AA–AF^	13.35 ± 0.34 ^O–U^	3.04 ± 0.61 ^Y–AB^	9.72 ± 0.70 ^O–S^
Kim 2-4	6.93 ± 0.44 ^X–AB^	10.63 ± 0.30 ^S–W^	8.10 ± 0.79 ^Q–U^	8.50 ± 0.03 ^P–T^
Kim 3-1	2.93 ± 0.17 ^AD–AH^	0.00 ± 0.00 ^AH AI^	1.21 ± 0.70 ^AA AB^	0.00 ± 0.00 ^AB^
Kim 3-2	13.99 ± 0.48 ^N–S^	19.85 ± 0.36 ^K L^	8.10 ± 0.79 ^Q–U^	9.72 ± 0.74 ^O–S^
Kim 4-1	24.75 ± 0.90 ^F–J^	15.18 ± 0.36 ^M–Q^	17.00 ± 0.64 ^I J K^	6.69 ± 0.33 ^S–Y^
Kim 4-2	0.00 ± 0.00 ^AH AI^	0.00 ± 0.00 ^AH AI^	0.00 ± 0.00 ^AB^	0.00 ± 0.00 ^AB^
Kim 4-4	0.00 ± 0.00 ^AH AI^	0.00 ± 0.00 ^AH AI^	3.65 ± 0.72 ^X–AB^	0.00 ± 0.00 ^AB^
Kim 5-1	0.00 ± 0.00 ^AH AI^	0.00 ± 0.00 ^AH AI^	0.00 ± 0.00 ^AB^	0.00 ± 0.00 ^AB^
Kim 5-4	3.28 ± 0.25 ^AC–AH^	4.20 ± 0.72 ^AB–AG^	4.45 ± 1.06 ^U–AA^	0.00 ± 0.00 ^AB^
Kim 6-1	12.68 ± 1.13 ^P–V^	5.60 ± 0.53 ^AA–AF^	9.72 ± 0.04 ^O–S^	0.00 ± 0.00 ^AB^
Kim 7-1	5.46 ± 0.55 ^AA–AF^	4.63 ± 0.58 ^AB–AG^	4.26 ± 0.37 ^U–AA^	0.00 ± 0.00 ^AB^
Kim 8-3	0.00 ± 0.00 ^AH AI^	0.00 ± 0.00 ^AH AI^	0.00 ± 0.00 ^AB^	0.00 ± 0.00 ^AB^
Kim 8-5	0.00 ± 0.00 ^AH AI^	0.00 ± 0.00 ^AH AI^	0.00 ± 0.00 ^AB^	0.00 ± 0.00 ^AB^
Kim 9-7	0.00 ± 0.00 ^AH AI^	0.00 ± 0.00 ^AH AI^	0.00 ± 0.00 ^AB^	0.00 ± 0.00 ^AB^
Kim 9-8	3.10 ± 0.85 ^AD–AH^	0.00 ± 0.00 ^AH AI^	0.00 ± 0.00 ^AB^	2.18 ± 0.43 ^Z–AB^
FR 1	2.39 ± 0.69 ^AF–AH^	5.14 ± 0.27 ^AB–AG^	0.00 ± 0.00 ^AB^	0.00 ± 0.00 ^AB^
FR 2	25.06 ± 0.99 ^F–I^	14.89 ± 0.49 ^M–Q^	21.28 ± 1.68 ^E–H^	15.12 ± 0.35 ^J K L^
HA	17.87 ± 0.42 ^L M^	6.02 ± 0.27 ^AA–AD^	14.97 ± 2.09 ^J K L^	3.23 ± 1.06 ^Y–AB^
HC	3.39 ± 0.65 ^AC–AH^	5.54 ± 0.48 ^AA–AF^	0.00 ± 0.00 ^AB^	0.00 ± 0.00 ^AB^

Values are presented as mean ± SEM. Within each column, means that share a letter do not differ significantly; means with different letters indicate a significant difference (*p* < 0.05).

**Table 3:** Basic genomic features of *L. paracasei* D2, *L. rhamnosus* JL, *W. cibaria* JLK, *L. sakei* F1.

Bacterial Strain	Genome Size (Mbp)	GC Content (%)	NCBI GenBank Accession Number
*L. paracasei* D2	3.36	45.8	PRJNA1251814
*L. rhamnosus* JL	2.96	46.6	PRJNA1252288
*W. cibaria* JLK	2.53	44.7	PRJNA1265932
*L. sakei* F1	1.98	41.0	PRJNA1093171

**Table 4:** Antioxidant-related gene comparison of strains D2, JL, JLK, and F1.

Genes Responsible for Antioxidant Activity in LAB	Gene Description	*L. paracasei* D2	*L. rhamnosus* JL	*W. cibaria* JLK	*L. sakei* F1
*npx*	NADH Peroxidase	+	+	+	+
*nox*	NADH Oxidase	−	+	−	−
*tpx*	Thiol Peroxidase	+	+	−	+
*trxB*	Thioredoxin Reductase	+	+	+	+
*gpx*	Glutathione Peroxidase	+	+	+	−
*gshR*	Glutathione Reductase	+	+	+	+
*sodA*	Superoxide Dismutase	+	−	−	+
*katE*	Catalase	−	−	−	+

(+) gene present; (−) gene absent.

**Table 5:** Upregulation of genes involved in antioxidant activity of strain *L. paracasei* D2 and *L. rhamnosus* JL at 2 mM H_2_O_2_ vs. Control.

System/Genes Responsible for Antioxidant Activity		Relative Expression Fold (D2)		Relative Expression Fold (JL)	
		Mid-Log	Start of Stationery	Mid-Log	Start of Stationery
NADH oxidase-peroxidase/	*npx*	1.42	0.78	1.44	0.94
	*nox*	-	-	1.25	1.07
Thioredoxin (Trx)/	*tpx*	1.23	0.96	1.26	0.85
	*trxB*	1.10	1.14	1.14	1.11
Glutathione peroxidase system/	*gpx*	0.86	1.13	1.28	1.24
	*gshR*	0.92	1.35	1.02	0.91

(-) gene is absent in the genome.

## Data Availability

The genome sequences of the isolates have been submitted to NCBI under the following accession numbers: *Latilactobacillus sakei* F1 (PRJNA1093171), *Lacticaseibacillus paracasei* D2 (PRJNA1251814), *Lacticaseibacillus rhamnosus* JL(PRJNA1252288), and *Weissella cibaria* JLK (PRJNA1265932).

## Abbreviations

The following abbreviations are used in this manuscript:

LAB	Lactic acid bacteria
H_2_O_2_	Hydrogen peroxide
ROS	Reactive oxygen species
BHA	Butylated hydroxyanisole
MBP	Metal-binding proteins
HK	Heat-killed
SOD	Superoxide dismutase
CAT	Catalase
